# T Rays vs. Terrorists: Widening the Security Spectrum

**DOI:** 10.1289/ehp.114-a540

**Published:** 2006-09

**Authors:** Valerie J. Brown

The 10 August 2006 arrest in Britain of 24 terrorists bent on smuggling bomb components aboard airplanes and combining them en route is just the latest salvo in the Darwinian battle between developers of terrorist weaponry and those seeking to defeat them. The array of diabolical methods available to terrorists is truly terrifying, ranging from nuclear weapons and “dirty bombs” to biological and chemical weapons and explosives.

Detection and assessment of terrorist threats is generally possible today with enough time, money, and laboratory equipment, but the ideal technology would be fast, accurate, cheap, easy to use, and portable or able to remotely detect threats, with an emphasis on prevention. No technology now exhibits all these virtues, but under the pressure of terrorists’ inventiveness, researchers are working steadily to develop and apply improved systems.

Now researchers at Argonne National Laboratory are getting promising results from experiments using “T rays,” the terahertz (THz) part of the electromagnetic spectrum. In March 2006 Argonne announced that a research team there had shown for the first time that T rays can be used to identify explosives and poison gas precursors. The Argonne team also successfully used millimeter-wave radar to remotely detect airborne chemicals and the effects of radiation in the air. These results are currently being written up for publication.

T rays and millimeter waves are at the low-energy end of the electromagnetic spectrum, between microwaves and infrared frequencies. According to Nachappa “Sami” Gopalsami, a senior electrical engineer at Argonne and a lead researcher on the THz sensor project, the general characteristics of T rays and millimeter waves are the same. “But,” he says, “new physics and phenomena are beginning to be explored as we move up in frequencies.”

Although many detection techniques currently in use are based on electromagnetic radiation and mass spectrometry, T rays and millimeter waves have not previously been used in this context, mainly due to an inability to generate broadband pulses in these frequencies. In the Argonne experiments, however, THz spectrometry sensors provided unambiguous identification of explosive chemicals, including TNT and plastic explosives. Gopalsami says this method is “highly specific” and will eliminate interference from confounding elements.

The Argonne team has been collaborating with researchers at Dartmouth College, Sandia National Laboratory, Sarnoff Corporation, and AOZT Finn-Trade of St. Petersburg, Russia. Funding has come from the U.S. Air Force, the Department of Energy, and the Department of Defense. But although national security imperatives are the driving force behind current research, many of the resulting technologies could also prove useful in environmental health applications.

Being able to remotely detect and identify chemicals will be helpful in monitoring gas pipeline leaks, chemical plants, vehicle emissions, and the like. Gopalsami says the T ray technology can detect some of the most important environmental hazards including ozone, volatile organics, and cyanide compounds. Medical applications, particularly imaging techniques for body tissues and teeth, are also in the offing, especially because the THz zone is on the opposite end of the electromagnetic spectrum from X rays, and thus of lower energy and far less damaging to living tissue.

## Detection Difficulties

Technical problems plague many existing detection methods. For example, X rays can penetrate almost anything but can harm the object being studied, and in living organisms they may damage DNA and cause cancer. Laser and other optical instruments are less harmful, but their performance can be affected by wind, humidity, fog, and smoke.

Just tracking terrorists’ movements is a nightmare. In a paper presented at the March 2002 Conference on Technology for Preventing Terrorism, David Dye of Lawrence Livermore National Laboratory noted that the United States has 7,606 miles of land border and some 12,452 miles of coastline. Further, Dye reported, 633.7 million people entered the United States at the nation’s 361 ports of entry. Even in the months just after September 11, the Coast Guard boarded only about 35% of the 5,112 vessels entering U.S. ports. Wrote Dye, “The government simply cannot perform 633.7 million hand searches every year, no matter how great the threat.”

“Our biggest concern is explosives,” says Nico Melendez, a spokesman for the Transportation Security Administration (TSA). Melendez says the TSA started airport screening for explosives using what’s called an “air shower” system in the summer of 2004. In this system, passengers step into a booth-like portal that releases puffs of air aimed at their clothing and skin. An air sample is then collected and analyzed by an ion mobility spectrometer, which compares the air’s components against a database containing spectrographic profiles of target chemicals such as TNT, C-4, and Semtex. According to a 24 May 2006 press release from the Port of Portland (Oregon), 28 airports in the United States are now using air shower portals.

THz waves are also useful for passenger screening because they can penetrate beneath clothing to detect hidden weapons. Peter Adrian, a senior analyst with business consultancy Frost & Sullivan, says, “One of the historical problems with gas sensors [including ion mobility spectrometers] is that they can be affected by extraneous environmental factors.” Conventional mobility spectrometers searching for explosives and trace levels of chemical warfare agents can’t always pick the target signal out of the “noise” of the many other chemicals in the environment, such as perfumes, and may be susceptible to false positives, causing delays and passenger frustration.

Faster and more accurate identification of questionable materials is crucial to effective protection from terrorism. With too many false positives, people will become desensitized to the danger. At the same time, a false negative means the system has failed, with potentially devastating consequences. The TSA is currently funding Argonne research into replacing the ion mobility spectrometer with THz spectrometry, says Gopalsami, who adds that with proper funding the device could be taken into the field in two years.

## Putting T Rays to the Task

Argonne’s THz spectrometry technology measures the rotation of a molecule in the vapor or gas phase. Every molecule’s rotational pattern is unique, and exciting a molecule with T ray frequencies reveals the “fingerprint” for that molecule. A spectral identification algorithm uses the information to determine the specific compound being examined by matching it with a spectral library. One disadvantage of THz spectrometry, says Gopalsami, is that to be detected a molecule must be polar, or asymmetrical; methane, for example, cannot be detected this way because it is nonpolar, or symmetrical.

Quick and accurate identification of a molecule is easiest when the molecules are rotating unimpeded in gas or vapor form under pressures well below normal atmospheric pressure, so that collisions between molecules are decreased. This is easy to establish in a laboratory, but difficult in field conditions. However, the Argonne researchers were able to overcome this handicap with millimeter-wave frequencies, which are less sensitive to atmospheric conditions; their longer wavelengths (relative to cloud particles) cause less reflection and scattering of the millimeter waves. “Additionally,” says Gopalsami, “there are gaps or windows in the millimeter-wave spectrum in which common molecules in air are mostly transparent to the millimeter waves.” Using millimeter-wave frequencies, the team identified airborne poison gas chemicals from 60 meters away and chemicals related to nuclear weapons from 600 meters.

A major issue for counterterrorist sensor development is whether a sensor must have a physical sample or whether it can detect and analyze a substance at a distance. The former are called “point sensors,” and the latter are “remote” or “standoff” detectors. Chemical, biological, and explosive materials generally require a point sensor. However, in an experiment with AOZT Finn-Trade, the Argonne team was able to tell when a nuclear power plant 9 kilometers away was in operation or idle by measuring radiation-induced changes in the air around the plant. Those changes were observable using microwave radar, but the team is also experimenting with millimeter-wave radar to achieve higher sensitivity of detection.

Bioweapons also pose serious risks, and the development of sensors capable of rapid remote detection has been slow. The litany of known and possible biological agents is frightening, among them the viruses that cause smallpox, anthrax, plague, and Ebola hemorrhagic fever. Further, in an article in the 2006 special issue of *EMBO Reports*, authors Jonathan Tucker and Craig Hooper described how advances in protein engineering could make so-called fusion toxins another front-runner for terrorists. These custom-made “designer” poisons unite two or more naturally occurring toxins, such as ricin and botulinum, to create a toxin significantly more toxic than either parent. Not only that, but unless counterterrorist researchers can stay abreast of possible combinations, a fusion toxin could be invisible to a sensor looking for a match in a preexisting library.

For bioweapon detection, Argonne researchers are working on a sensor based on dielectric properties of molecules. Dielectric materials are nonconducting and exhibit a complex property called a dielectric constant that can be measured by resonator techniques. Furthermore, they resonate at particular frequencies. DNA appears to resonate strongly in the THz region; therefore, the dielectric approach may eventually enable early detection of biological molecules without the use of more complex and much slower biochips that rely on analytical tools such as polymerase chain reaction.

As new technologies are developed, they will not necessarily eliminate older methods. “It’s hard to make a categorical statement that one approach is better than the others,” says Dye. Because the range of terrorist weapons is so broad, he adds, “You’ll end up with niche applications.” In the swirl of national security challenges, however, using a new part of the electromagnetic spectrum offers rich promise for thwarting the terrorist arsenal—and likely will produce benefits for environmental health as well.

## Figures and Tables

**Figure:**
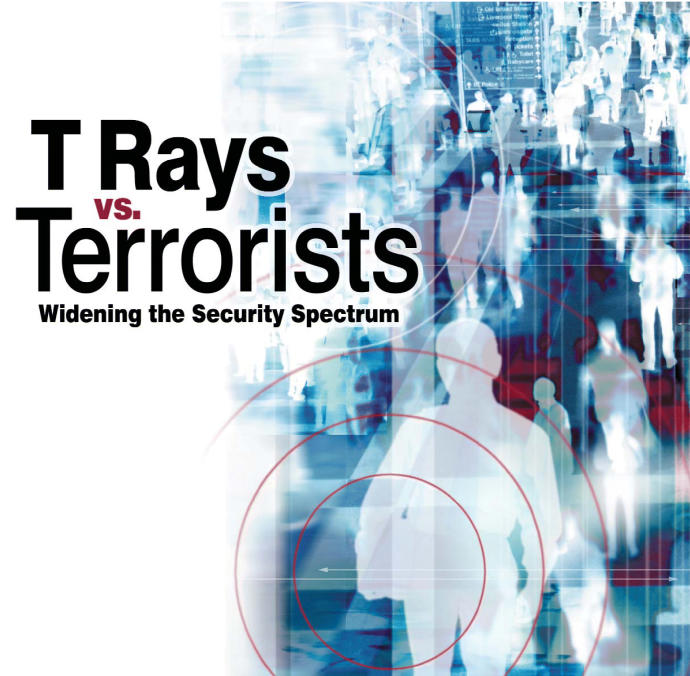


**Figure:**
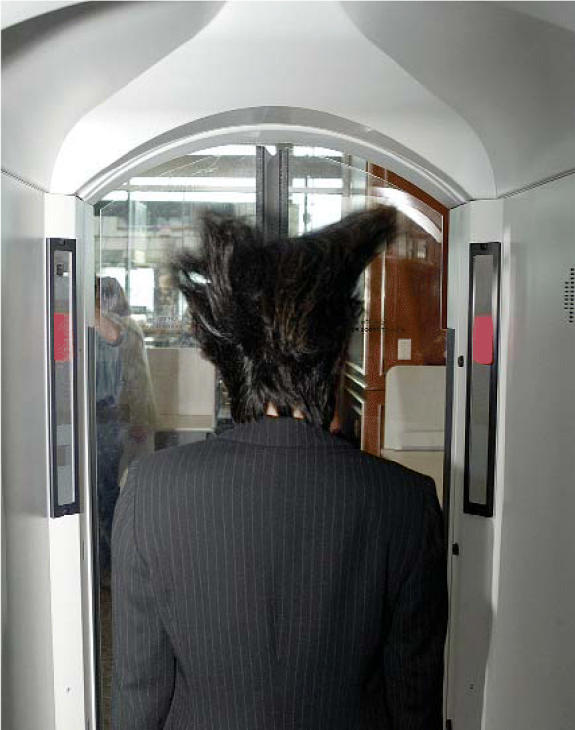
Hair-raising experience? One technology now used for airport security comprises portals in which puffs of air are blown at passengers and then analyzed to detect trace amounts of explosives. THz waves add the advantage of penetrating clothing, and offer a high probability of detection with few false alarms.

**Figure:**
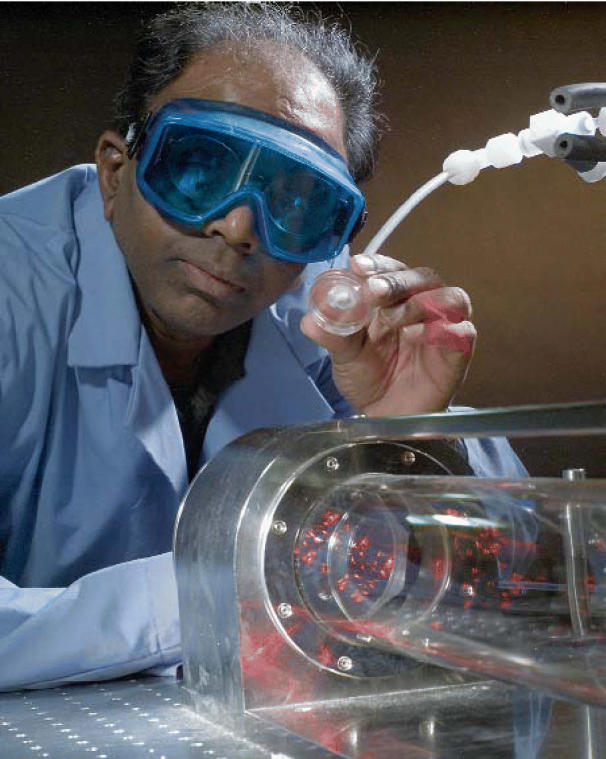
One in a billion Sami Gopalsami demonstrates an instrument in Argonne’s Terahertz Test Facility that can detect chemicals at the part-per-billion level.
